# Development and validation of a 3D printed antiviral ventilator filter - a comparative study

**DOI:** 10.1186/s12871-021-01310-z

**Published:** 2021-04-14

**Authors:** Ruth Shaylor, Mathew Francis, Esther Shaylor, Solomon Dadia, Barak Cohen

**Affiliations:** 1 Division of Anesthesia, Intensive Care, and Pain Management, Tel-Aviv Medical Center; Tel-Aviv University, 6 Weizmann Street, 6423906 Tel-Aviv, Israel; 2 Department of Food Science, University of Leeds, Leeds, UK; 3 Supply Division, United Nations Children’s Fund, Copenhagen, Denmark; 4 Surgical 3D printing Laboratory, Tel-Aviv Medical Center, Tel-Aviv, Israel; 5 Department of Orthopedic Oncology, Tel-Aviv Medical Center, Tel-Aviv University, Tel-Aviv, Israel; 6 Outcomes Research Consortium, Cleveland Clinic, Cleveland, OH USA

**Keywords:** COVID-19, 3D printing, Anesthesia, Ventilator, Global Health

## Abstract

**Background:**

The current coronavirus infectious disease 2019 (COVID-19) pandemic has caused unexpected pressure on medical supplies, interrupting supply chains and increasing prices. The supply of antiviral filters which form an essential part of the ventilator circuit have been affected by these issues. Three-dimensional (3D) printing may provide a solution to some of these issues.

**Methods:**

We designed and tested 3D printed heat and moisture exchange (HME) and antiviral casing. For each casing we tested two different filter materials derived from a sediment water filter cartridge or 1.5-μm glass fiber filter paper. A polyurethane sponge was used for the HME. Each design was tested for circuit leak, circuit compliance, peak inspiratory pressure and casing integrity using methylene blue dye.

**Results:**

We designed, produced, and tested two different types of antiviral filters with six different internal configurations. Overall, we tested 10 modified filter designs and compared them with the original commercial filter. Except for the combination of 1.5-μm filter paper and 5 mm sponge peak inspiratory pressure and circuit compliance of the filters produced were within the operating limits of the ventilator. All In addition, all filters passed the dye test.

**Conclusions:**

Our filter may be of particular importance to those working in low middle-income countries unable to compete with stronger economies. Our design relies on products available outside the healthcare supply chain, much of which can be purchased in grocery stores, hardware stores, or industrial and academic institutions. We hope that these HMEs and viral filters may be beneficial to clinicians who face critical supply chain issues during the COVID-19 pandemic.

**Supplementary Information:**

The online version contains supplementary material available at 10.1186/s12871-021-01310-z.

## Background

The recent coronavirus infectious disease 2019 (COVID-19) pandemic caused by the severe acute respiratory syndrome coronavirus 2 (SARS-CoV-2) originated in China in late 2019, and has since spread to most of the world, affecting millions and overwhelming health systems globally. Aside from the extreme effects on global economy and healthcare, the pandemic also caused an unexpected surge in demand of certain health-related supplies, unmet by existing supply chains [1, 2]. In particular items related to respiratory support and mechanical ventilation are among those in high demand.

This has led to an unprecedented amount of bartering with suppliers and export bans for certain items [3, 4]. Countries with relatively small purchasing power or those considered to be of low and middle income (LMIC) have been “priced out” of the market.

Filters form an essential part of the breathing circuit of mechanical ventilators, protecting their internal parts from various pathogens. In the intensive care unit (ICU) they are usually replaced every 24 h or earlier if they become clogged. In the operating room (OR) they should be replaced after each case. A filter rated for bacterial pathogens is normally sufficient. Under the current circumstances, however, the filter material needs to be dense enough to protect against viral pathogens and small aerosolized particles [5].

Three-dimensional (3D) printing has drawn attention as a potential temporary solution due to its flexibility and reproducibility. Once an item has been developed as an electronic stereolithography (STL) file it can be produced anywhere in the world with access to the right type of 3D printer. This has enabled hospitals and universities to print many items in house as well as an army of volunteers who have a home desktop 3D printer. The majority of these efforts have focused on personal protective equipment (PPE) or the development or modification of ventilators [6–8]. To date, there have been no media reports or publications on the development of 3D printed antiviral filters for use on anesthesia machines or ventilators, despite them forming an essential component in the care of COVID-19 patients.

Tel Aviv Medical Center (TLVMC) has gained experience in using 3D modelling and printing on perioperative planning [9–11]. Considering possible difficulties in acquiring antiviral filters during the current pandemic, this experience was utilized to meet the potential shortage, as a last life-saving resort in case of commercial supply-chain failure.

We hereby describe our experience in designing, developing, producing, and testing of an alternative 3D-printed viral filter for use in ventilator breathing circuits. We also share the specific files needed to reproduce our design, and the specific materials we used and tested. Lastly, we describe the methodology and results of the comparative validation tests we used to verify the feasibility of using our printed filter, compared to commercially available models.

## Methods

This was a single center feasibility study utilizing international multidisciplinary collaboration aimed at comparing the technical characteristics of various air filter designs. No patients were involved, and ethical review board was therefore deemed unnecessary.

### Filter material

After discussion with a filtration engineer (MF) it was decided to test a sediment cartridge normally used in home water filtration systems (Noga Watercare B.V., The Netherlands) rated at 5 μm, and 1.5 μm glass fiber filter paper (934-AH, Whatman, GE Healthcare, UK). The outer layer of the filter was peeled off, leaving a strip 1 mm thick. Using an existing heat and moisture exchange (HME) filter (Intersurgical, UK) that had been carefully taken apart as a template, a circle of the filter material was cut. This was then placed inside the existing HME and testing was carried out as described below.

Some of the required materials were easily available from retail stores. However, 1.5 μm (μm) filter paper was not available in stores or through local distributors. Eventually, we were able to receive this item from a local water treatment facility (Ayalon Water Treatment Plant, Ramla, Israel).

### Heat moisture exchange material

The foam in the commercial HME is 6 mm thick and has a similar density to commercially available polyurethane sponges (Scotch-brite, 3 M, USA) [12]. These were purchased from a local vendor and the abrasive part was removed. Using the HME as a template, discs of approximately 5 mm and 2 mm thickness were cut. The sponge discs were subsequently inserted into a commercial HME filter instead of the original sponge and tested on an anesthesia machine for inspiratory pressure, circuit compliance and circuit leak as described below.

### 3D design

A commercial HME filter (Intersurgical, UK) was measured using a computerised calibre (Fujifilm, Japan) by design engineers (Synergy3DMed, Netanya, Israel). These measurements were then transferred to a computer assisted design program (SP5.0 SolidWorks 2019, Dassault Système, USA) to build the model. After building the digital model, it was adapted to print on Stratasys J750 “Objet” printer (Stratasys, Israel) that allows simultaneous printing of multiple materials. The design was printed on the high mix setting using the biocompatible polymer Med610 filament (Stratasys, Israel). A similar process was repeated to design a 3D model of a commercial viral filter (Intersurgical, UK), that has no HME capabilities.

### Circuit compliance and leak testing

Discs of the various filter materials were placed inside the viral filter casing and inspiratory pressure, circuit compliance, and circuit leak were tested. A similar process was conducted for the HME but with the sponge inserts as well as the filter material. The materials used were compared to a commercially available HME filter at each stage of the filter design for circuit leak and compliance. All tests were carried out on the same Perseus A500 anesthesia machine (Drager, Lubeck, Germany).

The following tests were performed:

- The adjustable pressure valve (APL) was set to 30cmH_2_O. Using the auto leak test function, the filter was tested for circuit leak and circuit compliance.
- The APL was then opened.
- The filter was then attached to a 2000 ml test lung and the ventilator was set to a tidal volume of 520 ml, respiratory rate 12 breaths per minute and positive end-expiratory pressure (PEEP) of 3cmH_2_0. Gas flow was decreased to 1 l per minute. Peak inspiratory pressures were recorded.

The following combinations were evaluated:

- Commercial HME
- Commercial HME case, sediment filter cartridge
- Commercial HME case, 1.5 μm filter paper
- Commercial HME case, 5 mm sponge
- Commercial HME case, 2 mm Sponge
- 3D printed HME, Sediment filter cartridge, 5 mm sponge
- 3D printed HME, Sediment filter cartridge, 2 mm sponge
- 3D printed HME, 1.5 μm filter paper, 5 mm sponge
- 3D printed HME, 1.5 μm filter paper, 2 mm sponge
- 3D printed viral filter, sediment filter cartridge
- 3D printed viral filter, 1.5 μm filter paper

All tests were conducted 3 times for each combination, and the “worst” results are reported.

### Internal filter integrity testing

**Fig. 1:**
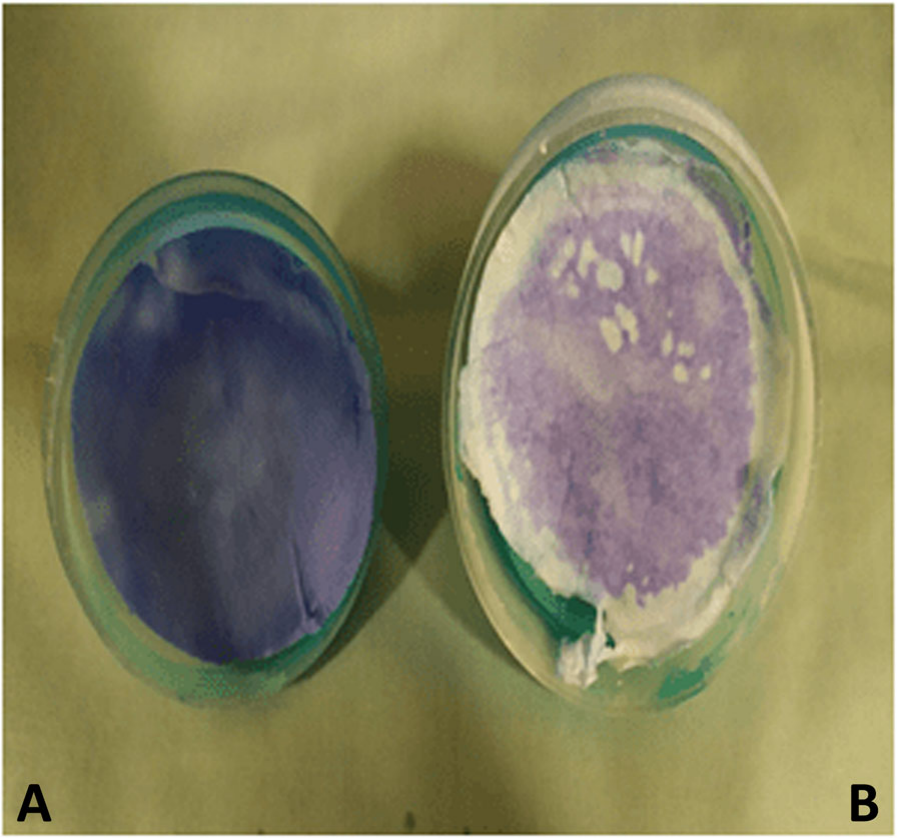
Illustration of the dye leak test. **a** 1.5 μm filter paper with dye leaking around the edges. **b** 1.5 μm filter paper where all the dye has passed through the filter material without leaking around the edges

To confirm the integrity of the filter, a transfer medium was used to determine if any voids were present in the completed filter. Aqueous methylene blue (Sterop NV, Belgium) was diluted to a concentration of 0.05 mg/ml and used as a dye. Fifty ml of the dye were passed through the filter from the expiratory side. The exterior of the filter was visually examined for leaks. Following the test, the filter was taken apart and the spread of the dye on the filter material was visually inspected. If a clear margin was observed on the filter paper where the dye had not spread to parts of the filter casing, it was assumed that the transfer medium had passed through the filter and not around it [13, 14]. Therefore, it was assumed that filters which passed this test would have air flowing through the filter paper once connected to the anesthesia machine (Fig. 1).

## Results

**Fig. 2:**
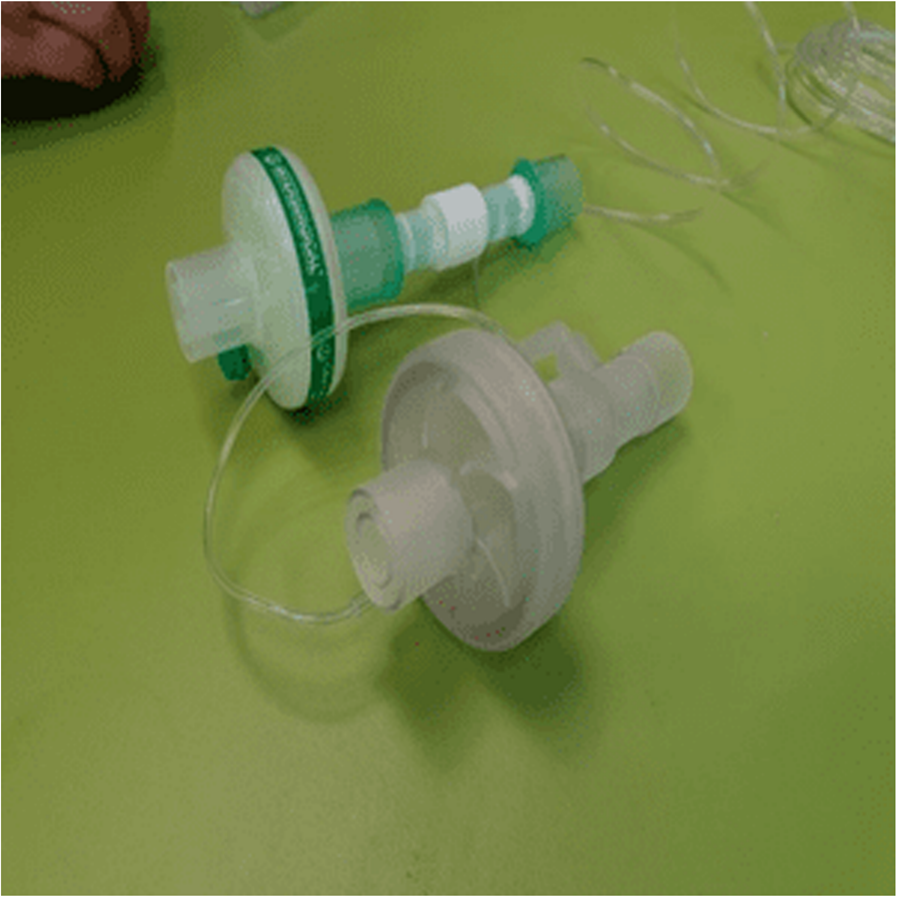
The commercial (upper side, Intersurgical, UK) and 3D printed HME filters

We were able to design, produce, and test two different types of antiviral filters with six different internal configurations (Fig. 2). Overall, we tested 10 modified filter designs and compared them with the original commercial type. The results of the static pressure leak test, compliance, and peak pressures, as well as the dye leakage test, are presented in Table 1.

**Table 1:** Test results of 10 filter designs from leak tests performed on a Perseus A500 anesthesia machine (Drager, Lubeck, Germany)

Device	Circuit leak (ml/min)	Circuit Compliance (ml/cmH_**2**_O)	Peak inspiratory pressure (cmH_**2**_O)	Adequate dye test
**Commercial HME**	10	1.3	19	NA
**Commercial HME, sediment filter cartridge**	10	1.4	23	NA
**Commercial HME, 1.5** μm **filter paper**	44	1.5	19	NA
**Commercial HME, 5 mm sponge**	43	1.4	21	NA
**Commercial HME, 2 mm Sponge**	80	1.5	20	NA
**3D printed HME, Sediment filter cartridge, 5 mm sponge**	69	1.4	20	Yes
**3D printed HME, Sediment filter cartridge, 2 mm sponge**	70	1.4	19	Yes
**3D printed HME, 1.5 μm filter paper, 5 mm sponge**	15	1.4	27	Yes
**3D printed HME, 1.5 μm filter paper, 2 mm sponge**	35	1.5	22	Yes
**3D printed viral filter, sediment filter cartridge**	67	1.4	20	Yes
**3D printed viral filter, 1.5 μm filter paper**	29	1.4	23	Yes

*Leak* compliance and peak pressure tests were conducted 3 times, and the wort result is reported, *3D* three-dimensional, *HME* heat and moisture exchanger, *NA* not applicable

The maximal leak among all combinations was 80 ml/min, which is well below the upper allowed limit of the anesthesia station examined (150 ml/min). The circuit compliance did not change significantly compared to the reference filter (maximal compliance of 1.5 ml/cmH_2_O compared to the 1.3 ml/cmH_2_O reference). Similar results were found for the peak inspiratory pressure, with a maximum of 27 cmH_2_O compared to a reference of 19 cmH_2_O. Of note, the 3D printed filters did not result in any variable outside the acceptable range [15]. All filters passed the dye test.

## Discussion

We successfully designed and tested an antiviral and HME filter with 3D printed casing in 1 week. We produced two versions of each filter using different filter materials. Except for the combination of 1.5 μm filter paper and 5 mm sponge, which had an unacceptably high peak inspiratory pressure, both produced circuit leak and compliance tests within the normal operating limits of the anesthesia machine they were tested on [15].

Healthcare systems around the globe are currently experiencing unforeseen demand of medical items. To mitigate the impact of this shortage on LMICs we used simple materials accessible outside the normal hospital supply chain to successfully develop and test 3D printed HMEs and viral filters for use on anesthesia machines and ventilators. These casings have been trialed to resemble the properties of those available commercially.

COVID-19 spreads via aerosolization and contact with contaminated surfaces [16]. Aerosols occur when solid or liquid molecules are dispersed through air. Their size can range from 0.3–100 μm in diameter. Particles of 1–5 μm remain in the air and therefore, present the biggest challenge [5]. Considering this method of transmission, appropriate filtration of ventilator circuits is of vital importance [17].

The antiviral properties of 1.5 μm filter paper have been previously investigated [18, 19]. Whilst more effective at trapping larger particles (1.5 μm) they are effective at trapping particles as small as 0.3 μm, particularly at highly humid environments as found in the HME [5, 20]. The 1.5 μm filter paper is usually used by water processing plants, and in food and drink manufacturing. As a relatively specialist material, it may not be readily available.

The antiviral properties of the 5 μm sediment cartridge have been less well investigated. The pore size of this material seems too large. Nevertheless, this is the pore size when the material is saturated with water. When used in air, the pore size is one-tenth the size in water [21]. This would provide a pore size of 0.5 μm, which should be sufficient. It is also more widely available and easier to source, in case the 1.5 μm filter paper is unavailable. The product we used is also available as a 1-μm sediment cartridge which is suitable as an antiviral filter [22].

The humidifying properties of a foam-based HME depend on the foam’s density. Humidification capability is proportional to density, but it increases resistance to air flow. The density of the foam used in the commercial HME is 26–32 kg m^− 3^ [23]. We calculated the density of the foam we used gravimetrically as 21 kg m^− 3^. The porosity of the foam was calculated as 98% which is in line with previous studies of polyurethane foams [24, 25]. It is not unreasonable, therefore, to expect that both foams will produce similar humidification at standard operating room conditions.

Another potential advantage to our filter is that it can be disassembled and the 3D printed parts re-sterilized using either commercially available wipes or by soaking in bleach. Once dry, the filter and HME material can be replaced and it can be reused.

We have completed the feasibility and suitability tests demonstrating that the different filters comply with the anesthesia machine specifications. However, we did not evaluate their actual antiviral, antibacterial, and heat and moisture preservation capabilities and further investigations in this area should be considered prior to use. Nevertheless, all designs passed the methylene-blue dye test demonstrating that the dye passed through the filter rather than around it. This is similar to most 3D printed N95 masks reported in the literature which have at best undergone leak testing but no further clinical evaluation.

It should be pointed out, however, that these filters are only intended for use as a last resort to overcome an abrupt interruption of the supply chain, rather than to replace commercial alternatives. As a class II medical device, we would strongly encourage anyone planning on using our design to apply for an Emergency Use Approval from their relevant regulatory body.

Whilst production of designs in the healthcare setting suitable for 3D printing requires a certain amount of technical skills, reproducing them in the field does not [26, 27]. With mobile data being nearly ubiquitous, an internet connection is no longer required in order to share models with colleagues working in low resource or rural settings. STL files are relatively small (2–70 megabytes [Mb]), and can be printed in geographically remote locations with no internet access using a mobile phone, laptop, SD card, and a 3D printer. The whole process requires about 600 Mb of data storage. We have also printed these filters using polylactic acid (PLA) and polyvinyl alcohol (PVA) filler on a desktop Makerbot Method printer (Stratasys, Minnesota, USA). One cartridge of PLA will make approximately 9–13 filters. The STL file we produced is available as Supplemental Material to this report.

In these tumultuous times, it is a responsibility of the international medical community to provide assistance to our colleagues who are experiencing shortage of equipment, either through a disrupted supply chain or by being priced out of the market. We hope that these HMEs and viral filters may be of use to clinicians who may face critical supply chain issues during the COVID-19 pandemic.

## Supplementary Information

**Additional file 1.**

## Data Availability

The datasets used and/or analysed during the current study are available from the corresponding author on reasonable request.

## Abbreviations

3D: Three-dimensional
COVID-19: Coronavirus infectious disease 2019
HME: Heat and moisture exchange
ICU: Intensive care
LMIC: Low and middle income country
OR: Operating room
PEEP: Positive end-expiratory pressure
PLA: Polylactic acid
PPE: Personal protective equipment
PVA: Polyvinyl alcohol
SARS-CoV-2: Severe acute respiratory syndrome coronavirus 2
STL: Stereolithography
TLVMC: Tel Aviv Medical Center
